# Neuraminidase in Virus-like Particles Contributes to the Protection against High Dose of Avian Influenza Virus Challenge Infection

**DOI:** 10.3390/pathogens10101291

**Published:** 2021-10-07

**Authors:** Hae-Ji Kang, Ki-Back Chu, Keon-Woong Yoon, Gi-Deok Eom, Jie Mao, Min-Ju Kim, Su-Hwa Lee, Eun-Kyung Moon, Fu-Shi Quan

**Affiliations:** 1 Department of Biomedical Science, Graduate School, Kyung Hee University, Seoul 02447, Korea; haedi1202@naver.com (H.-J.K.); ckb421@gmail.com (K.-B.C.); kgang92@gmail.com (K.-W.Y.); ekd3910@naver.com (G.-D.E.); maojie@khu.ac.kr (J.M.); mj16441@naver.com (M.-J.K.); dltnghk228@nate.com (S.-H.L.); 2 Department of Medical Zoology, School of Medicine, Kyung Hee University, Seoul 02447, Korea; ekmoon@khu.ac.kr; 3 Medical Research Center for Bioreaction to Reactive Oxygen Species and Biomedical Science Institute, School of Medicine, Graduate School, Kyung Hee University, Seoul 02447, Korea

**Keywords:** avian influenza, virus-like particle, neuraminidase, protection

## Abstract

Neuraminidase is an important target for influenza vaccination. In this study, we generated avian influenza VLPs, expressing hemagglutinin (HA), neuraminidase (NA), HA and NA co-expressed (HANA), to evaluate the protective role of NA against a high (10LD_50_) and low (2LD_50_) dose of avian influenza virus challenge infections. A single immunization with HANA VLPs elicited the highest level of virus-specific IgG, IgG1, and IgG2a responses from the sera post-vaccination and the lungs post-challenge-infection. Potent antibody-secreting cell responses were observed from the spleens and lungs of HANA-VLP-immunized mice post-challenge-infection. HANA VLPs induced the highest CD4^+^ T cell, CD8^+^ T cell, and germinal center B cells, while strongly limiting inflammatory cytokine production in the lungs compared to other VLP immunization groups. In correlation with these findings, the lowest bodyweight losses and lung virus titers were observed from HANA VLP immunization, and all of the immunized mice survived irrespective of the challenge dose. Contrastingly, VLPs expressing either HA or NA alone failed to elicit complete protection. These results indicated that NA in VLPs played a critical role in inducing protection against a high dose of the challenge infection.

## 1. Introduction

Highly pathogenic avian influenza viruses such as the H5N1 subtype are a major threat to humans and other animals. As evidenced by the 1997 Hong Kong pandemic, direct transmission of the avian influenza virus to humans is possible, and the resulting outcome can be fatal [1]. On a global scale, culling countless numbers of birds due to avian influenza outbreaks in poultry farms incurred economic losses ranging in the billions [2]. Vaccines are the most effective measures for preventing influenza outbreaks, and efforts to develop broadly protective influenza vaccines are still ongoing. Despite these extensive endeavors, avian influenza vaccines were reported to be less efficacious and less immunogenic than trivalent inactivated influenza vaccines. Even upon an increased immunization dosage or adjuvant use, antibody seroconversion rates of avian influenza vaccines were comparable to those of trivalent inactivated vaccines [3]. Another factor hindering efficacious avian influenza vaccine development is the emphasis placed on hemagglutinin (HA) antigens in the vaccine design strategy [4]. Moreover, because humans are thought to possess minuscule levels of pre-existing immunity to the conserved domains of HA, it was hypothesized that avian HA-based vaccines would be weakly immunogenic [5]. Therefore, an alternative strategy must be employed to develop an efficacious avian influenza vaccine.

Virus-like particles (VLPs) are highly immunogenic vaccine platforms and efficacies forms of VLP-based vaccines are actively being investigated against a wide array of infectious diseases, including avian influenza. To date, several studies demonstrated that avian influenza HA-expressing VLP vaccines can induce protection [6,7,8,9]. Neuraminidase (NA) is another glycoprotein expressed on the surface of influenza viruses and a strong correlation between the NA antigen and protection can be drawn [10]. Immunizing mice with the NA antigen generated using the computationally optimized broadly reactive antigen (COBRA) methodology was reported to be capable of enhancing the breadth of protection against diverse influenza strains, thus paving the way to developing a successful universal influenza vaccine [11]. Nevertheless, NA antigens continue to remain largely undermined in vaccine design strategies [4]. Currently, only a few VLP-based vaccine studies involving avian NA antigens have been conducted [12,13,14]. However, while these aforementioned studies delineated that VLPs co-expressing both HA and NA are efficacious, many of the NA-VLP-induced immune correlates associated with protection were not investigated.

Infection dose is another factor that must be taken into consideration in vaccine studies, as evidenced by the presence of a strong correlation between infection dose and disease severity for influenza viruses. For instance, experimentally infecting mallards and chickens with increasing embryonic infective doses (EID_50_) of avian influenza virus resulted in increased mortality in these birds [15]. An identical trend was also observed in CD1 mice, where increasing the infection dose led to enhanced mortality regardless of the virus inoculation route [16]. In the case of the human influenza virus, it was hypothesized that the higher case fatality during the second and third waves of the 1918 H1N1 influenza pandemic was possibly attributed to higher infectious doses arising from a greater proportion of infected individuals [17], and that the duration of viral shedding is closely related to disease severity [18]. Currently, the role of NA-antigen-expressing vaccines and their correlation with the challenge dose remains unreported. Therefore, investigating this aspect of NA-based vaccines against high and low challenge infection doses could have a significant impact.

Here, to demonstrate that complementing vaccines with NA is crucial for enhancing protection against avian influenza virus infection, VLP vaccines expressing the HA, NA, or both antigens of H5N1 avian influenza were generated for immunization studies. We compared the protective efficacies of VLPs expressing either HA or NA alone to those of HA and NA co-expressing VLPs against high (10LD_50_) or low (2LD_50_) infection doses. Findings presented here provide vital information for the avian influenza vaccine design strategy that can be utilized to control avian influenza outbreaks.

## 2. Results

### 2.1. Gene Cloning and VLP Characterization

**Figure 1:**
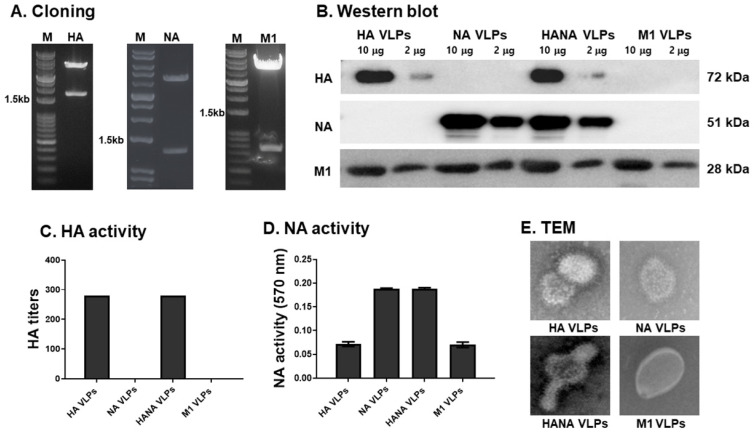
Successful gene cloning and VLP generation were confirmed through multiple characterization assays. (**A**) HA (1695 bp), NA (1368 bp), or M1 (759bp) genes inserted into pFastBac confirmed by restriction digestion analysis. (**B**–**E**) VLPs were characterized using western blot, HA activity, NA activity, and TEM. HA and NA activity data are presented as mean ± SD. Abbreviations: HA VLPs—virus-like particles (VLPs) expressing hemagglutinin (HA) antigen only; NA VLPs—VLPs expressing neuraminidase (NA) antigen only; HANA VLPs—VLPs expressing both HA and NA antigens; M1 VLPs—VLPs expressing only M1 antigen; TEM—transmission electron microscopy.

Hemagglutinin (HA), neuraminidase (NA), and matrix 1 protein (M1) genes were cloned into the pFastBac vector and cleaved with restriction enzymes. Gel electrophoresis results revealed that the genes were correctly inserted into vectors (Figure 1A), and these were subsequently used to generate VLPs using the baculovirus expression system. VLPs were characterized using western blot, HA and NA activity, as well as transmission electron microscopy (TEM). HA, NA, and M1 components of the VLP constructs were detected using western blot (Figure 1B). HA antigens were only detected from HA and HANA VLPs, whereas NA antigen expression was only observed from NA and HANA VLPs. M1, which was used as a structural core protein for the VLPs, was detected from all VLP constructs. HA activities were measured using the HA titer assay, and these were only observed from VLPs expressing the HA antigen (Figure 1C). For the NA activity assay, positive OD_570nm_ values were only observed from VLPs expressing NA antigens, while HA and M1 readings were basal levels (Figure 1D). The morphological analysis and successful generation of the VLPs were confirmed by TEM (Figure 1E).

### 2.2. Virus-Specific Antibody Responses from the Sera of Immunized Mice

**Figure 2:**
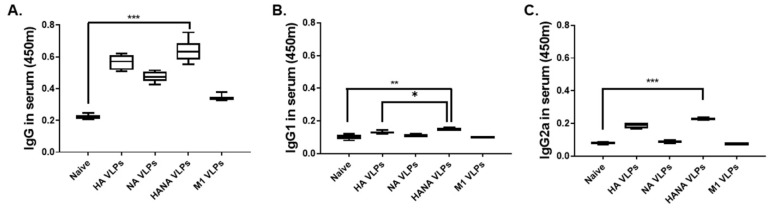
Virus-specific antibody responses were detected from the sera of mice (*n* = 6) 4 weeks after immunization. IgG (**A**), IgG1 (**B**), and IgG2a (**C**) responses were confirmed by ELISA. Data are presented as median ± SD (* *p* < 0.05, ** *p* < 0.01, *** *p* < 0.001) and are representative of the three independent experiments performed in triplicates. Abbreviations: HA VLPs—virus-like particles (VLPs) expressing hemagglutinin (HA) antigen only; NA VLPs—VLPs expressing neuraminidase (NA) antigen only; HANA VLPs—VLPs expressing both HA and NA antigens; M1 VLPs—VLPs expressing only M1 antigen.

Mice were immunized once with the VLPs, and sera were collected 4 weeks after the immunization. To confirm successful immunization, sera of immunized mice were reacted with the inactivated A/Chicken/Vietnam/G04/2004 (H5N1) virus, and influenza virus-specific antibody responses were measured. A single immunization with influenza VLPs was capable of eliciting IgG antibody responses, with VLPs expressing both HA and NA inducing the strongest response (Figure 2A). IgG1 antibody responses were more or less similar across all groups, but a significant increase was only observed from the HANA VLPs (Figure 2B). NA and M1 VLPs failed to elicit virus-specific IgG2a responses, while VLPs displaying the HA antigen mounted IgG2a antibody responses (Figure 2C).

### 2.3. VLP Immunization Induces Strong Antibody Responses against Lethal Doses of Influenza Virus Challenge Infection

**Figure 3:**
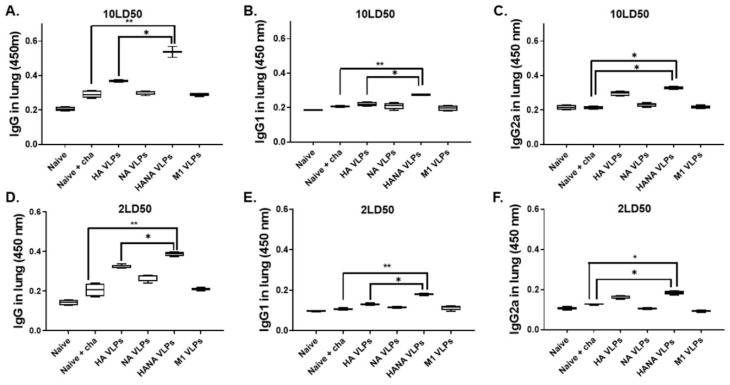
Lung samples (*n* = 3) were collected from challenge-infected mice, and antibody inductions were evaluated. (**A**–**C**) IgG, IgG1, and IgG2a responses of mice infected with 10 LD_50_ influenza virus were determined by ELISA. (**D**–**F**) Antibody levels in the lungs of 2 LD_50_ virus-infected mice were assessed. Data are presented as median ± SD (* *p* < 0.05, ** *p* < 0.01) and are representative of the two independent immunization studies conducted on mice. Abbreviations: Naïve + cha—unimmunized infection control; HA VLPs—virus-like particles (VLPs) expressing hemagglutinin (HA) antigen only; NA VLPs—VLPs expressing neuraminidase (NA) antigen only; HANA VLPs—VLPs expressing both HA and NA antigens; M1 VLPs—VLPs expressing only M1 antigen.

Antibody responses from the lung homogenates of challenge-infected mice and their contribution to protection were assessed. The IgG antibody was significantly enhanced in HANA-VLP-immunized mice challenged with 10 LD_50_, whereas the responses were comparable across the remaining groups (Figure 3A). A similar trend was observed for both IgG1 and IgG2a (Figure 3B,C). As with the 10 LD_50_ challenge-infection dose, antibody inductions were strikingly similar in mice challenged with 2 LD_50_. While potent IgG responses were observed from HANA VLPs and also from HA VLPs to some extent, the antibody levels were similar across the remaining groups (Figure 3D). This phenomenon was also observed for IgG1 and IgG2a responses (Figure 3E,F).

### 2.4. Single Immunization with the VLPs Elicited Strong Antibody-Secreting Cell (ASC) Responses in Mice

**Figure 4:**
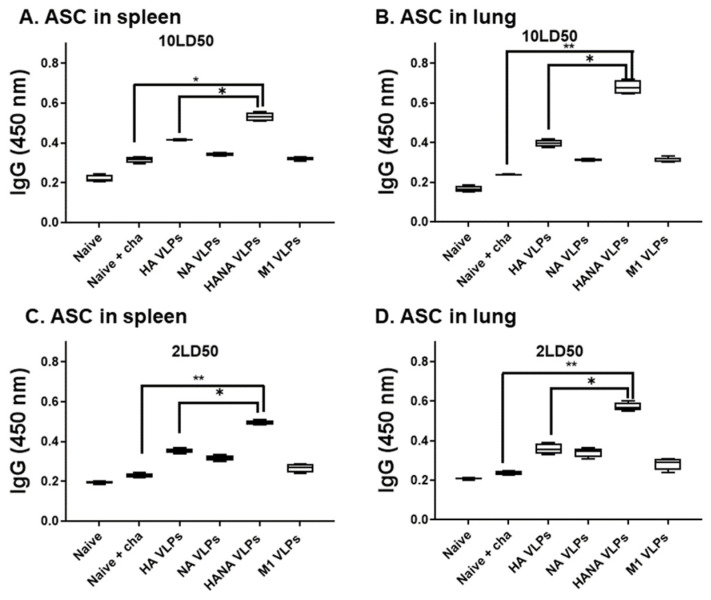
Spleen and lung samples (*n* = 3) were collected from challenge-infected mice, and ASC responses were evaluated. (**A**,**B**) ASC responses in the spleens and lungs of mice infected with 10 LD_50_ influenza virus were determined by ELISA. (**C**,**D**) ASC responses of mice infected with 2 LD_50_ virus were assessed from the spleen and the lung. Data are presented as median ± SD (* *p* < 0.05, ** *p* < 0.01) and are representative of the two independent immunization studies conducted on mice. Abbreviations: Naïve + cha—unimmunized infection control; HA VLPs—virus-like particles (VLPs) expressing hemagglutinin (HA) antigen only; NA VLPs—VLPs expressing neuraminidase (NA) antigen only; HANA VLPs—VLPs expressing both HA and NA antigens; M1 VLPs—VLPs expressing only M1 antigen.

ASC responses from the spleen and lungs of mice were evaluated post-challenge with the 10 or 2 LD_50_ influenza virus. Results were similar to antibody responses observed from sera and lung homogenates. The strongest IgG ASC responses were detected from spleens (Figure 4A) and lungs (Figure 4B) of mice immunized with the HANA VLPs upon the 10 LD_50_ challenge infection. This trend was also observed from the spleens and lungs of the 2-LD_50_-challenged mice (Figure 4C,D).

### 2.5. VLPs Induced Robust Cellular Imune Responses in Mice

**Figure 5:**
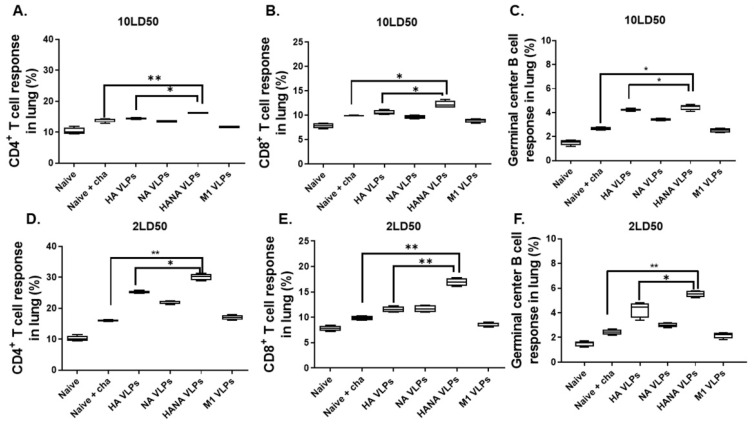
Lung samples (*n* = 3) were homogenized, and single cell populations were prepared for flow cytometry analyses. (**A**–**C**) CD4^+^ T cell, CD8^+^ T cell, and GC B cells were analyzed from the lungs of mice infected with 10 LD_50_ avian influenza virus. (**D**–**F**) CD4^+^ T cell, CD8^+^ T cell, and GC B cells were analyzed from the lungs of mice infected with 2 LD_50_ avian influenza virus. Data are presented as median ± SD (* *p* < 0.05, ** *p* < 0.01) and are representative of the two independent immunization studies conducted on mice. Abbreviations: Naïve + cha—unimmunized infection control; HA VLPs—virus-like particles (VLPs) expressing hemagglutinin (HA) antigen only; NA VLPs—VLPs expressing neuraminidase (NA) antigen only; HANA VLPs—VLPs expressing both HA and NA antigens; M1 VLPs—VLPs expressing only M1 antigen.

Cellular immune responses were detected from the lungs of mice. After the challenge infection with 10 LD_50_ of the avian influenza virus, the highest CD4^+^ and CD8^+^ T cell populations were observed from the lungs of HANA-VLP-immunized mice (Figure 5A,B). Germinal center B (GC B) cell populations were comparable between HA-VLP- and HANA-VLP-immunized groups (Figure 5C). Similarly, HANA-VLP-induced T cells were significantly elevated compared to naïve-challenge controls when infected with the 2 LD_50_ dose of the avian influenza virus (Figure 5D,E). GC B cell inductions were similar as well, with the highest induction being observed from HANA VLPs, followed by HA VLPs and NA VLPs (Figure 5F).

### 2.6. Single Immunization with the VLP Vaccines Suppresses Pulmonary Inflammation

**Figure 6:**
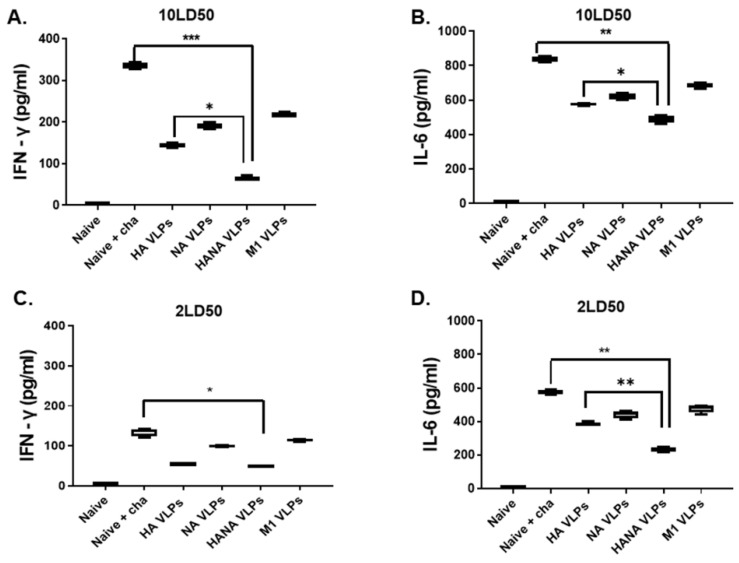
Cytokine productions in the lung homogenates (*n* = 3) were quantified. (**A**,**B**) IFN-γ and IL-6 productions in the lung samples were assessed following 10 LD_50_ challenge infection. (**C**,**D**) IFN-γ and IL-6 productions in the lung samples were quantified using cytokine ELISA after 2 LD_50_ challenge infection. Data are presented as median ± SD (* *p* < 0.05, ** *p* < 0.01, *** *p* < 0.001) and are representative of the two independent immunization studies conducted on mice. Abbreviations: Naïve + cha—unimmunized infection control; HA VLPs—virus-like particles (VLPs) expressing hemagglutinin (HA) antigen only; NA VLPs—VLPs expressing neuraminidase (NA) antigen only; HANA VLPs—VLPs expressing both HA and NA antigens; M1 VLPs—VLPs expressing only M1 antigen.

Production of the pro-inflammatory cytokines interferon-gamma (IFN-γ) and interleukin 6 (IL-6) was assessed from the lung homogenates of challenge-infected mice. Compared to the naïve + cha control, HANA VLPs significantly diminished the production of IFN-γ upon the 10 LD_50_ challenge infection. In other immunization groups, IFN-γ levels were not significantly different from the naïve + cha group (Figure 6A). Consistent with this finding, HANA VLPs lessened the IL-6 production, while marginal reductions were observed from HA, NA, and M1 VLPs (Figure 6B). The challenge infection with 2 LD_50_ also resulted in similar results, with a marked reduction in IFN-γ and IL-6 being observed from HANA-VLP-immunized mice (Figure 6C,D).

### 2.7. A Single VLP Immunization Lowers Lung Virus Load and Contributes to Protection

**Figure 7:**
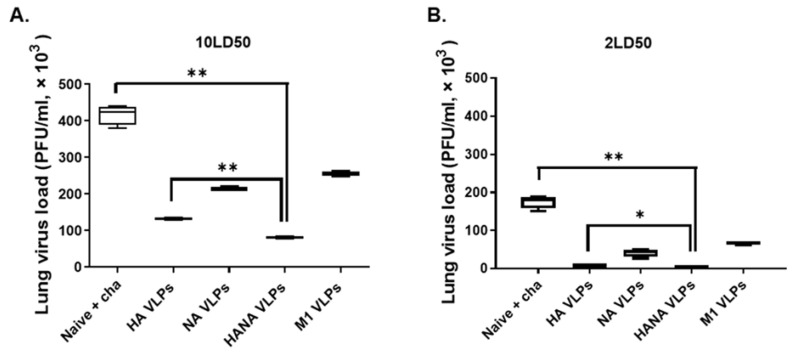
Lung tissues (*n* = 3) were collected from challenge-infected mice, and plaque assay was performed to quantify lung virus titer. (**A**) Lung homogenates of VLP-immunized mice challenged with 10 LD_50_. (**B**) Virus titers of mice challenge-infected with 2 LD_50_. Data are presented as median ± SD (* *p* < 0.05, ** *p* < 0.01) and are representative of the two independent immunization studies conducted on mice. Abbreviations: Naïve + cha—unimmunized infection control; HA VLPs—virus-like particles (VLPs) expressing hemagglutinin (HA) antigen only; NA VLPs—VLPs expressing neuraminidase (NA) antigen only; HANA VLPs—VLPs expressing both HA and NA antigens; M1 VLPs—VLPs expressing only M1 antigen.

Plaque assays were performed to assess the lung virus loads of challenge-infected mice. All of the VLPs significantly lessened the virus burden in the lungs when challenged with the 10 LD_50_ dose of the avian influenza virus. (Figure 7A). Compared to the naïve + cha control, the greatest reductions in lung viral loads were observed from HANA VLPs. For mice challenged with the low dose of the avian influenza virus, similar results were found. HANA greatly diminished the viral replications in the lungs which were comparable to those elicited by HA VLPs. (Figure 7B). Overall, a single immunization with the VLPs reduced the lung viral burden in mice irrespective of the challenge infection dose.

### 2.8. NA Antigen Is Critical in Inducing Protection against High Challenge Dose Infection

**Figure 8:**
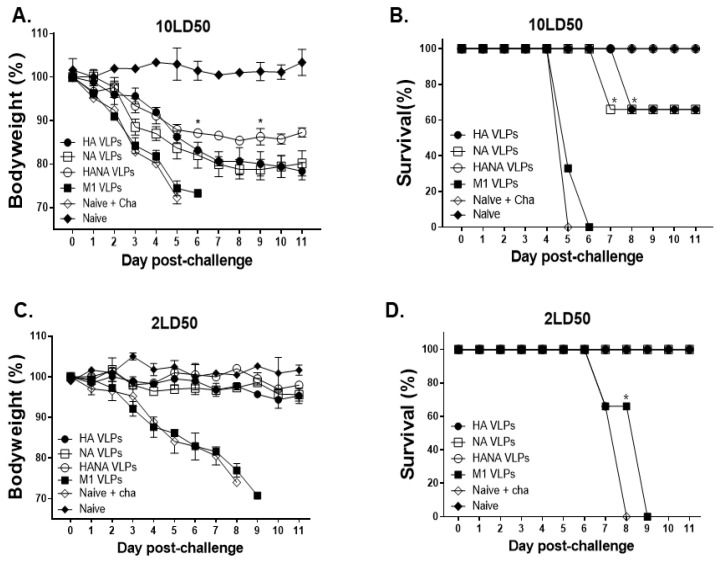
Bodyweight reductions and survival of mice (*n* = 6) were monitored for 11 days post-challenge infection with high and low doses of avian influenza virus. (**A**,**B**) After challenge infection with 10 LD_50_, bodyweight and survival were measured. (**C**,**D**) Bodyweight reduction and survival data of low dose challenge infection (2 LD_50_). Data are presented as mean ± SD (* *p* < 0.05) and are representative of the two independent immunization studies conducted on mice. Abbreviations: Naïve + cha—unimmunized infection control; HA VLPs—virus-like particles (VLPs) expressing hemagglutinin (HA) antigen only; NA VLPs—VLPs expressing neuraminidase (NA) antigen only; HANA VLPs—VLPs expressing both HA and NA antigens; M1 VLPs—VLPs expressing only M1 antigen.

Bodyweight reductions and the survival of challenge-infected mice were monitored for 11 days. M1 VLP immunization and naïve + cha mice were humanely euthanized around 5 days post-infection (dpi). Mice immunized with HA, NA, and HANA VLPs conferred protection, although significant bodyweight reductions were noticeable in all three immunization groups (Figure 8A). A single immunization with the HA and NA VLPs failed to confer protection against a high dose of the influenza virus challenge infection. In both groups, only 60% of the immunized mice survived, while all of the mice immunized with the HANA VLPs survived (Figure 8B). In the low dose challenge infection, naïve + cha and M1 mice were euthanized at 8 dpi onwards. Changes to bodyweight were negligible in HA-, NA-, and HANA-VLP-immunized mice, as all three groups maintained a close to normal bodyweight (Figure 8C). Unlike the high infection dose groups, HA-, NA-, and HANA-VLP-immunized mice were protected from the low dose challenge infection (Figure 8D).

## 3. Discussion

In this study, we generated VLPs expressing the HA and NA surface antigens of the avian influenza virus and assessed their protective efficacies against high and low doses of the lethal challenge infection. Our findings indicate that the protective efficacies between HA and NA VLPs were strikingly similar with minor differences in immunological parameters associated with protection, hence resulting in identical survival rates. However, VLPs co-expressing both HA and NA antigens elicited a higher degree of protection than VLPs expressing either antigen alone. Evidently, HANA VLPs elicited the highest influenza-virus-specific antibody responses and cellular immune responses. Moreover, inflammatory cytokine production and lung virus titers were the lowest in mice immunized with the HANA VLPs. This, by trend, contributed to the survival of HANA-VLP-immunized mice, while two out of three of the mice immunized with either HA or NA VLPs survived after the high dose homologous challenge infection.

Infections with various respiratory viruses such as influenza are frequently associated with a Th1 immune response induction [19]. However, different influenza vaccines appear to elicit a differing immune response dominance in animals. For example, vaccinating mice with the purified HA primarily induced the production of IgG1 which signifies Th2-dominance, whereas vaccination with VLPs or whole-inactivate virions evoked IgG2a and IgG2b subclasses indicative of Th1 immunity [20]. In accordance with this finding, BALB/c mice immunized in our study elicited stronger IgG2a responses than IgG1. In our study, the overall antibody responses were induced to a greater extent in mice immunized with the VLPs expressing HA antigens than those immunized with the NA VLPs. One major factor contributing to this result stems from CD4 T cell reactivity to the influenza virus antigens. For example, in H1N1-infected BALB/c mice, a slightly higher proportion of influenza-specific CD4 T cells were found to target the HA antigen than NA [21]. Conversely, CD4 T cell reactivity to NA antigens were much greater in C57BL/6 mice [21], thus implying the role of a host genetic background in immune response induction. Based on this rationale, a higher antibody response induction via HA VLPs was to be expected. The NA antigens of avian influenza VLP vaccines can enhance protection against a homologous challenge infection. For instance, increasing the NA contents in H5N1 VLP vaccines elicited protection exceeding those elicited by H5 VLPs, as indicated by enhanced survival and less bodyweight reduction [14]. Consistently, while bodyweight reductions were detected to an extent in the HANA VLPs, they were not as severe as those observed from HA or NA alone VLPs following a homologous challenge infection with the 10 LD_50_ avian influenza virus. Co-expressing HA and NA in VLPs also enhanced survival against a high challenge infection dosage, thus highlighting the importance of NA antigens for vaccine design.

To date, only a handful of experimental studies comparing the effect of an avian influenza virus inoculum dose have been conducted, but the findings appear to be consistent with our results. Varying levels of EID_50_ of the H5N1 virus were inoculated into chickens, and their histopathologies were assessed, with increasing the EID_50_ dose inflicting more inflammatory histopathologic lesions and resulting in less survival [22]. Similarly, the 10 LD_50_ H5N1 used here incurred greater pulmonary IFN-γ and IL-6 productions than 2 LD_50_. Both CD4^+^ and CD8^+^ T cells are highly regarded for their role in limiting influenza virus infection symptoms, as well as viral clearance in mice. One of the primary functions of CD8^+^ T cells is the active removal of virus-infected cells to suppress virus transmission [23]. Our study revealed that immunizing mice with the VLPs enabled the proliferation of CD4^+^ and CD8^+^ T cells in the lungs. In particular, the highest T cell inductions were observed from HANA-VLP-immunized mice, which exceeded those elicited by VLPs expressing either HA or NA alone. In this regard, enhanced CD8^+^ T cell proliferation induced by HANA VLPs likely contributed to lessening the lung virus titer. Several studies previously reported the upregulation of IL-6 expressions in the lungs of H5N1-infected patients [24,25]. Consistently, in both high and low dose infections with the H5N1, substantially high levels of IL-6 responses were detected even in immunized mice. However, their presence in the lung is important, and impaired IL-6 production may not necessarily be beneficial. In the absence of IL-6 or the IL-6 receptors, mice were reported to perish after infection with a sublethal dose of H1N1 [26]. As such, maintaining a balance between protection while minimizing inflammation incurred by IL-6 is of importance.

In avians, a single immunization with the oil-emulsified inactivated vaccines was reported to confer partial protection from the homologous H5N1 challenge infection [27]. However, when avians were immunized once with the recombinant H5 protein produced in mammalian cells, protection was observed, as indicated by the absence of virus shedding from the cloaca swabs or their trachea [28]. It is worth noting that protein subunit vaccines are subjected to misfolding and conformational instability, which can often result in poor immunogenicity [29,30]. VLPs, which are more immunogenic than subunit vaccines [29], could be useful to address such limitations. Evidently, a single immunization with the VLPs presented herein conferred protection against high and low homologous challenge infection doses. However, as the present study was conducted using mice, further validating our findings in avian models is required to confirm that supplementing the NA antigen along with HA confers better protection. 

## 4. Materials and Methods

### 4.1. Animals, Cells, and Viruses

Seven-week-old female BALB/c mice were purchased from NARA Biotech (Seoul, Korea). Animal experimental protocols were approved and conducted following the guidelines set out by the Kyung Hee University IACUC (permit number: KHUASP(SE)-18-024). *Spodoptera frugiperda* 9 (Sf9) cells were used to generate rBVs and VLPs. Madin–Darby canine kidney (MDCK) cells were used to perform plaque assays as previously described [31]. The avian influenza virus A/Chicken/Vietnam/G04/2004 (H5N1) was prepared as previously described [32].

### 4.2. Cloning of Avian Influenza Antigens and Generation of rBVs and VLPs

The H5, N1, and M1 genes of the A/Chicken/Vietnam/G04/2004 (H5N1) avian influenza virus were used to generate VLPs as previously described [33,34]. Briefly, genes were cloned into a pFastBac vector, and successful transformants were inserted into DH10Bac competent cells. After harvesting the bacmid DNA of transformants, they were transfected into Sf9 cells for rBV generation. For HA, NA, and HANA VLP generation, M1 rBVs were co-infected with HA rBV, NA rBV, and HA and NA rBVs, each respectively. At 3 days post-infection, culture media were harvested and centrifuged at 6000 RPM for 30 min. Supernatants were collected and subjected to ultracentrifugation at 30,000 RPM, 1 h, 4 °C. Pellets were resuspended in PBS and overlayed on top of the sucrose gradient (15%, 30%, 60%). Opaque bands were carefully collected, resuspended in PBS, and ultracentrifuged at 30,000 RPM, 1 h, 4 °C. Sedimented pellets were resuspended in PBS, and the protein assay was performed using the MicroBCA protein assay kit as per the manufacturer’s guidelines (ThermoFisher, Waltham, MA, USA). VLPs were stored at −80 °C until use. To confirm that the majority of the rBVs were removed from the VLPs, a monolayer of Sf9 cells was infected with the VLPs along with respective controls as described previously [35,36]. Cells were monitored daily for 4 days, and cellular infectivities were assessed under the microscope.

### 4.3. Characterization of VLPs

Western blotting was performed to confirm the expression of HA, NA, and M1 antigens (Figure S1). Proteins were loaded onto 10% sodium dodecyl sulfate-polyacrylamide gels and transferred onto a nitrocellulose membrane. Membranes were blocked with 5% skim milk prepared in Tris-buffered saline with 0.1% Tween-20 (TBST) at RT for 1 h. Blocked membranes were incubated with sera of mice infected with H5N1 (1:1000 dilution in PBS) to detect HA antigens. For NA and M1, an anti-NA monoclonal antibody (HCA-2, 1:10,000 dilution in PBS) and anti-M1 (GA2B, Abcam, Cambridge, UK) diluted 1:1000 in PBS were used, respectively. The pan-NA HCA-2 monoclonal antibody was kindly provided by Dr. Sang-Moo Kang, Georgia State University, GA, USA. Membranes were incubated with the primary antibodies overnight at 4 °C. After washing, membranes were probed with anti-mouse IgG (1:2000) or anti-rabbit IgG (1:10,000) secondary antibodies (Southern Biotech, Birmingham, AL, USA). Bands were developed with enhanced chemiluminescence, and images were acquired using the ChemiDoc imaging system (Bio-Rad, Hercules, CA, USA). VLP morphologies were confirmed using transmission electron microscopy (TEM). VLPs were adsorbed to sample grids and stained with 2% uranyl acetate. Images were acquired using the Bio-High voltage EM system (JEM-1400 Plus at 120 kV and JEM-1000BEF at 1000 kV, JEOL Ltd., Tokyo, Japan) at the Korea Basic Science Institute.

### 4.4. HA and NA Activity Assessment

HA activity was evaluated using the method previously described [37]. NA was measured in VLPs using the Amplex Red neuraminidase assay kit following the manufacturer’s instructions (Invitrogen, Waltham, MA, USA) as described elsewhere [38]. In brief, 10 μg of each VLP sample were diluted with 50 μL of the Amplex Red reaction buffer and added to a flat-bottom 96 well plate. Afterward, 50 μL of the Amplex Red working solution were inoculated into each well. Plates were incubated in the dark for 30 min at 37 °C, and OD_570nm_ values were measured using the EZ Read 400 microplate reader (Biochrom Ltd., Cambridge, UK).

### 4.5. Mouse Immunization and Challenge Infection

Seven-week-old female BALB/c mice (*n* = 6 per group) were intramuscularly immunized once with 10 μg of each of the VLPs. Sera were collected 4 weeks after immunization via a retro-orbital plexus puncture. At week 5 post-immunization, mice were challenge-infected with either 10 LD_50_ or 2 LD_50_ of the A/Chicken/Vietnam/G04/2004 (H5N1) avian influenza virus. At 4 dpi, 3 mice from each group were sacrificed for organ sampling and ex vivo studies, while the remaining 3 mice in each group were used to monitor bodyweight changes and survival until 11 dpi. In accordance with the institutional IACUC guidelines, mice that lost more than 25% of their initial bodyweight were humanely euthanized.

### 4.6. Virus-Specific Antibody Responses in Sera

Virus-specific antibody responses were detected using the enzyme-linked immunosorbent assay (ELISA) as previously described [34]. Briefly, inactivated A/Chicken/Vietnam/G04/2004 (H5N1) influenza virus antigens were coated in 96 well plates at a concentration of 4 μg/mL in carbonate coating buffer. Plates were blocked with 0.2% gelatin at 37 °C, 1 h. Sera collected from mice were diluted in PBS (1:100) and used as primary antibodies. After inoculating the sera into respective wells, plates were incubated at 37 °C, 1 h. Horseradish-peroxidase (HRP)-conjugated anti-mouse IgG, IgG1, and IgG2a secondary antibodies (1:2000 dilution in PBS) were added, and plates were incubated at 37 °C, 1 h. The O-phenylenediamine (OPD) substrate dissolved in substrate buffer with H_2_O_2_ was added for colorimetric assessment. OD_450nm_ values were measured using a microplate reader.

### 4.7. Pulmonary and Splenic Antibody-Secreting Cell (ASC) Responses

For ASC response, single cell populations of splenocytes (1 × 10^6^) and lung cells (5 × 10^5^) were prepared using a frosted microscope slide glass and Percoll gradient as previously described [39]. Briefly, splenocytes and lung cells were added to 96 well culture plates coated with 4 μg/mL of the inactivated A/Chicken/Vietnam/G04/2004 virus. Plates were incubated at 37 °C with 5% CO_2_. After 5 days, wells were washed and incubated with HRP-conjugated mouse IgG antibodies. The OPD substrate was added, and OD_450nm_ values were measured using a microplate reader.

### 4.8. Flow Cytometry, Cytokine Assays, and Lung Virus Titer

Single cell populations of lung cells were used to assess CD4^+^ T cell, CD8^+^ T cell, and GC B cell populations via flow cytometry as previously described [34]. Cells were stimulated with 0.5 μg/mL of the A/Chicken/Vietnam/G04/2004 H5N1 virus at 37 °C, 2 h. Afterward, cells were stained with fluorophore-conjugated CD3, CD4, CD8, B220, GL7, CD19, and IgD antibodies (BD Biosciences, Franklin Lakes, NJ, USA). An Accuri C6 flow cytometer was used for cell acquisition, and populations were analyzed using the Accuri C6 software (BD Biosciences, Franklin Lakes, NJ, USA). Expressions of the pro-inflammatory cytokines IFN-γ and IL-6 were assessed from the homogenized lung extracts using the BD OptEIA ELISA kit (BD Biosciences, Franklin Lakes, NJ, USA). A plaque assay was performed to quantify lung virus titers by infecting the confluent monolayer of MDCK cells with murine lung extracts as previously described [40].

### 4.9. Statistical Analysis

The statistical analysis was performed using GraphPad Prism 5 software (GraphPad Software Inc., San Diego, CA, USA). Significance was determined using one-way and two-way analysis of variance (ANOVA) with Bonferroni’s post hoc test. Data are expressed as either median ± SD or mean ± SD, and statistical significance between groups was denoted using asterisks (* *p* < 0.05, ** *p* < 0.01, *** *p* < 0.001). The entire immunization experiments were performed twice, while all other serological assays were performed three times in triplicates.

## 5. Conclusions

In summary, our findings elucidated that combining NA antigens with HA confers better protection than either antigen expressed alone on VLP vaccine platforms. Although the current vaccine design strategy is skewed towards HA, new vaccine formulation incorporating NA could drastically improve vaccine efficacy and pave the way towards a universal influenza vaccine.

## Data Availability

Data supporting the findings of this study are contained within the article.

## Supplementary Materials

The following are available online at https://www.mdpi.com/article/10.3390/pathogens10101291/s1, Figure S1: Confirming successful expression of HA, NA, and M1 in the VLPs.
