# Supplementary material for: Neuraminidase in Virus-like Particles Contributes to the Protection against High Dose of Avian Influenza Virus Challenge Infection
Source: Pathogens. 2021 Oct 7;10(10):1291. doi: 10.3390/pathogens10101291 (PMC8537550; doi:10.3390/pathogens10101291)
Supplement: Supplementary file 1 [file pathogens-10-01291-s001.zip › pathogens-1358826-supplementary.pptx]

## Slide 1
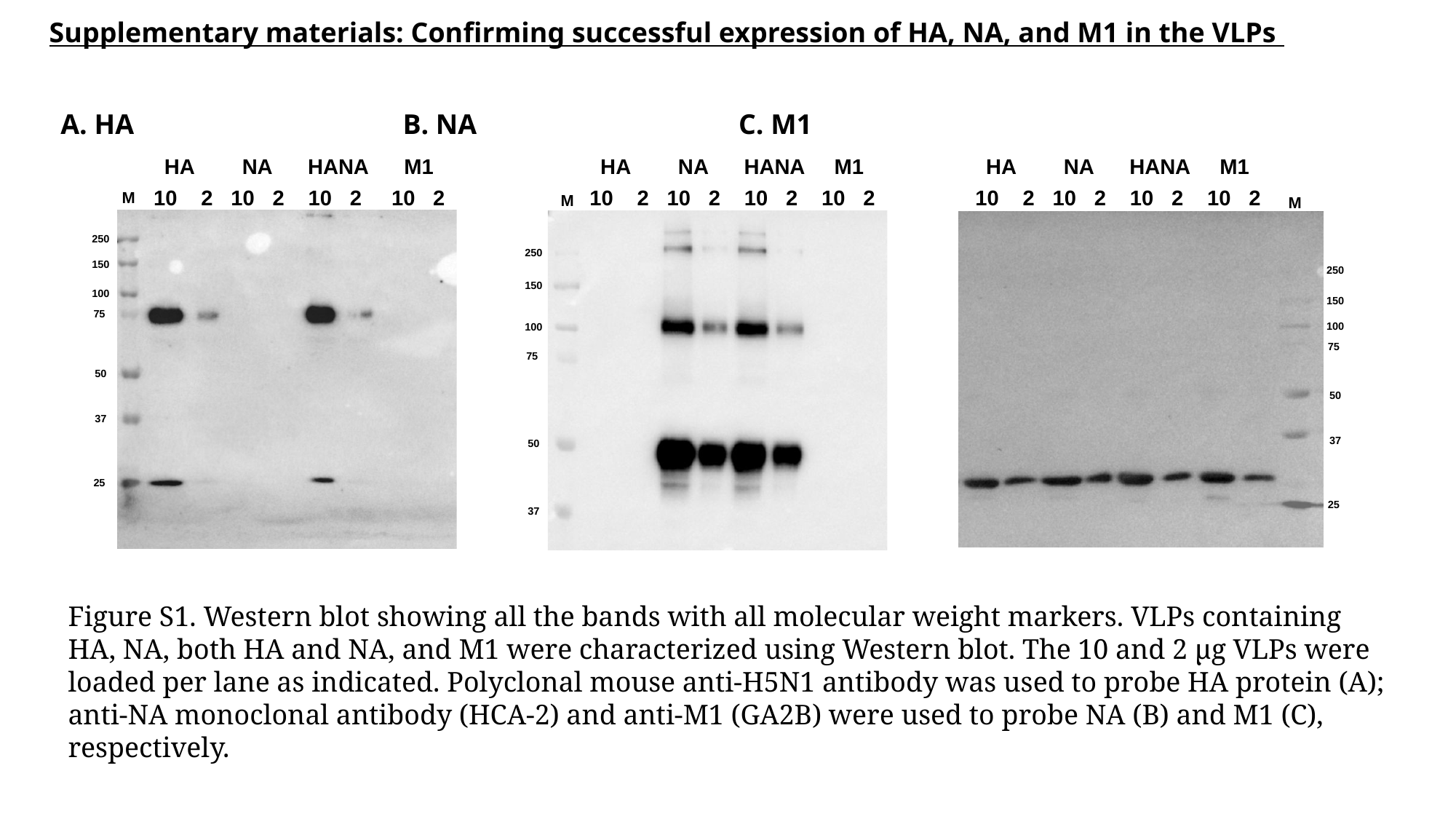

Supplementary materials: Confirming successful expression of HA, NA, and M1 in the VLPs
A. HA B. NA C. M1
HA NA HANA M1
HA NA HANA M1
HA NA HANA M1
 10 2 10 2 10 2 10 2
 10 2 10 2 10 2 10 2
 10 2 10 2 10 2 10 2
M
M
M
250
150
100
75
50
37
25
250
150
100
75
50
37
250
150
100
75
50
37
25
Figure S1. Western blot showing all the bands with all molecular weight markers. VLPs containing HA, NA, both HA and NA, and M1 were characterized using Western blot. The 10 and 2 μg VLPs were loaded per lane as indicated. Polyclonal mouse anti-H5N1 antibody was used to probe HA protein (A); anti-NA monoclonal antibody (HCA-2) and anti-M1 (GA2B) were used to probe NA (B) and M1 (C), respectively.
